# Patterns, Types, and Outcomes of Head Injury in Aseer Region, Kingdom of Saudi Arabia

**DOI:** 10.1155/2019/2782146

**Published:** 2019-03-07

**Authors:** Ibrahim Alnaami, Shbeli Alshehri, Saeed Alghamdi, Meshal Ogran, Asim Qasem, Abdulrahman Medawi, Abdulmajeed Medawi, Saud Alshahrani, Leen Sarhan

**Affiliations:** ^1^Neurosurgery Section, Department of Surgery, King Khalid University, Aseer Central Hospital, Abha, Saudi Arabia; ^2^Department of Surgery, King Khalid University, Abha, Saudi Arabia

## Abstract

**Background:**

Head injuries contribute to almost 50% of all injuries. Head injuries are still one of the major causes of loss of life and loss of function among young adults. Nowadays, head injury has become a major community problem. Recently, head injury has become one of the biggest issues of almost more than 57 million people in the whole world living with the neurological problem raised by TBI, in which 10 million people require hospital base care.

**Objectives:**

To determine the epidemiological aspects of patients with head injury (HI) in Aseer Central Hospital (ACH).

**Materials and Methods:**

This is a retrospective cross-sectional study. Data were gathered from patients' files and the registrar's database of ACH. The study duration was January 2015–December 2017. All patients with head injury admitted to ACH during the study duration were included in the study. SPSS software was used for analysis. Descriptive statistics were obtained (mean SD frequencies, percentages). Statistical tests, t test, and chi-squared test were applied to measure the significant difference among the variables. P-value less than 0.05 was considered as a significant difference.

**Results:**

There were 353 patients with head injury, and the mean ± SD of age was 27.01 ± 13.9. Motor vehicle accidents (MVA) accounted for (89.3%) of head injury. A total of 87.3% of the patients were male while 12.7% were female.

**Conclusion:**

In this study, we observed that MVA is the leading cause of brain/head injuries in the KSA, despite the implementations of new speed rules. However, with new regulations of forbidding cell phone use while driving and forcing the seat belt regulations, a major impact on these numbers is expected in the future. Thus, a future study is recommended to assess these expectations.

## 1. Introduction

Head injuries contribute to almost 50% of all injuries. Head injuries are a major cause of loss of life and loss of organs among young adults [1–4].

Nowadays, head injury has become one of the biggest issues of almost more than 57 million people in the whole world living with the neurological problem raised by TBI, in which 10 million people require hospital base care [5].

Head injuries are significant causes of deaths and disability irrespective of age groups. In light of the epidemiological findings from the last ten years, some effective preventive measures were planned, such as the most appropriate health care provision for both acute care and rehabilitation of disabled survivors [6]. Head injury accounted for 2/3 of inhospital trauma deaths. Estimated epidemiology data depicted that the frequency of TBI is higher in North America and Europe. On average, 2.8 million people had a TBI annually [6]. Head injury also affected the economy of the countries, produced some financial losses, and reduced the productivity. Almost US$60 billion was used to overcome the damages of HI in year 2000 [7, 8]. The estimated population incidence of traumatic brain injury in the United States was 73.5/100,000. A US-based study reported that head injuries were most common among young children [9, 10]. In the year of 1998 in Malaysia, 4.75% emergency patients were suffering from head injuries [11]. One epidemiology study stated that 69 million individuals worldwide were estimated to suffer from TBI [12].

Based on an Ethiopian study, head injuries are more common in males than in females. Deaths are positively correlated with severe head injuries in all age groups. Head injury was mild in the majority of head injury victims, followed by severe and moderate based on the Glasgow Coma Scale (GCS) score [13].

According to a Nigerian study, head injury was observed to be the most common among all injuries [14].

Saudi population is estimated to be 33,920,622, according to February 2019 United Nations estimates. Among 1,870 MVA victims in KSA, 30% of them died as a result of the accident. A further alarming finding was that most patients (56.7%) had head injuries [15].

According to another study from KSA, 32.1% of 1,219 patients suffered head injuries and MVAs were the leading cause of head injuries (34.2%) [16].

## 2. Objectives

The objective is to determine the epidemiological aspects of patients with head injury (HI) in Aseer Central Hospital (ACH), which holds one of the highest numbers of car accidents based on the census of the Ministry of Interior, KSA.

## 3. Materials and Methods

This is a retrospective cross-sectional study. Data were gathered from patients' files and the registrar's database of the ACH. The study duration was January 2015–December 2017. All patients with head injury admitted to ACH during the study duration were included in the study.

The variable included demographic data, Glasgow coma score, Glasgow outcome score, type of head injury, mechanism of injury, surgery type, and disposition of patients. Data were entered in the SPSS ver. 20 software for analysis. Descriptive statistics were obtained (mean SD frequencies, percentages). Statistical tests, t test, and chi-squared test were applied to measure the significant difference among the variables. P-value less than 0.05 was considered as a significant difference.

## 4. Results

**Figure 1:**
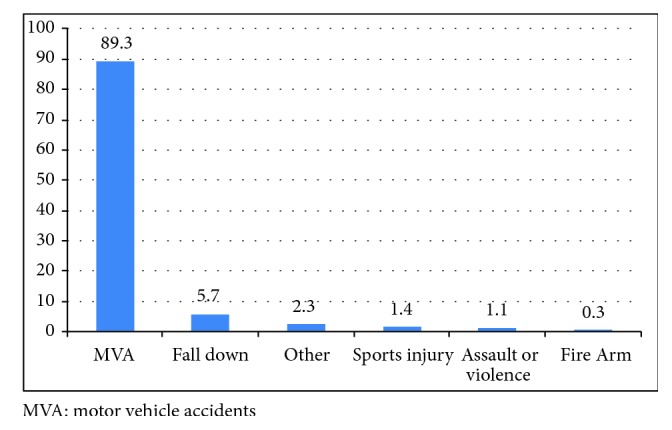
Mechanism of head injuries (n=353).

**Figure 2:**
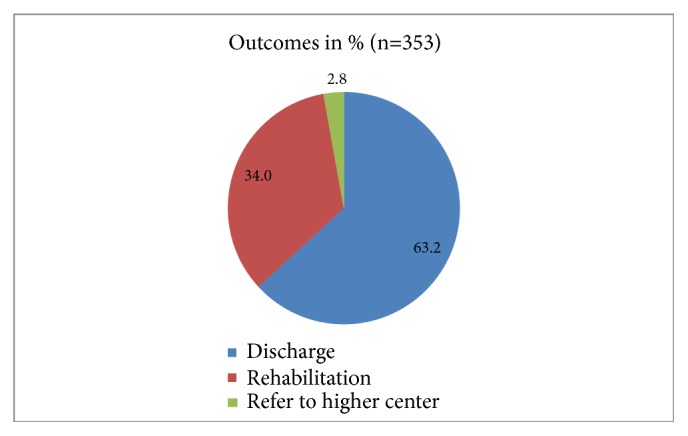
Disposition of head injury patients.

Out of 353 patients with head injury, we observed that the mean ± SD of age was 27.01 ± 13.9.  Figure 1 showed that MVA (89.3%) is the most leading cause of head injury. A total of 87.3% of the patients were male, while 12.7% were female; 94% were Saudi nationals, while 6% were foreign nationals; 55.3% resided in high altitudes (mountain areas), 38.3 in low altitudes (plain), and 8.4% in others; 42.5% were employed; 15.9% were unemployed; 34.6% were students; and 4% were workers (Table 1).  Figure 2 depicted that 34% went to rehabilitation centers, 63.2% were discharged, and 2.8% were referred to other centers during the acute phase based on either family'/patient's request or patient being from another province.

A total of 46.7% had severe GCS scores I (GCS<=8), 42.2% had moderate scores, and 11.1% had mild scores (Table 2). Based on Table 3, 2.5% of the patients died, while 64.3% had good recovery.  Table 4 showed that we did not observe any significant difference between Glasgow outcome score and head injury types, although subdural and intraventricular hemorrhages tend to have lower scores on Glasgow outcome score.  Table 5 shows that there was a significant difference between type of head injury and GCS scores (P<0.05), as patients with subdural hematomas and patients with brain contusions are noticed to have Glasgow coma score upon presentation lower than patients who have other head injury types. In Table 6, it is clearly shown that there is a significant difference between type of head injuries and outcomes in terms of placement at the end of acute management (p=0.0001), where patients with intraventricular hemorrhages and subdural hemorrhages tend to be placed in rehabilitation service more than patients with other head injury types.  Table 7 shows that patients with subdural hematoma were undergoing craniectomy if they were to get operated on; otherwise they tend to be treated medically. In regard to patients with traumatic subarachnoid hemorrhage, almost 30% of them were undergoing craniectomy, of course not for the subarachnoid itself, however, due to major underlying brain edema. Craniectomies were less likely to be done in patients with brain contusions or epidural hematomas (P<0.05).

## 5. Discussion

Our aim was to discuss the epidemiological aspects of patients with head injury (HI) in ACH, Abha, KSA. The occurrence of head injuries refers to the number of new cases recognized in a certain period. Almost each year under study approximately 1.7 million head injury/brain injury cases were recorded in the United States (in all age groups), and it is a contributing factor in approximately 30.5% of deaths related to injuries. Some studies showed the likelihood of brain injury being found more, in the babies and toddlers (0 to 4 years), adolescents from 15 to 19 years, and matures adults having age of 65 years or more [17].

According to a Malaysian study, MVAs were the common cause of head injury worldwide, after accidents at home, workplace, and during a sports event. In this study, 10% of patients were referred to higher centers, 29% went through the rehabilitation process, and 68% were discharged. Based on the findings of the Malaysian study, head injury was one of the increasing (7.86%) causes of hospitalization in Malaysian government hospitals in 2014 [18].

In one review of 26 studies (Tagliaferri et al.), traumatic brain injury (TBI) is the common cause of most trauma deaths in European countries [19], that is, 235 /100,000 patients with a mean mortality of 15/100,000 patients per year. In our study, MVAs were the major cause of head injuries, which is comparable with other studies. For example, one study reflected that, in five European countries, traffic accidents were the major (47%) cause of head injuries [20].

In this study, there were 87.3% male and 12.7% female; another study in Saudi Arabia described that males were more affected with head injury than females (78.4% vs. 21.6%) [21]. These results were also comparable with those of Jason Kisser (2017) [22]. The results indicated that men are 2.4 times more often to sustain a TBI in their lifetime than women. The Glasgow Coma Scale (GCS) score, after its introduction in 1974 [23], has been frequently used as one of the most important predictors of outcome after head injury. In our study, based on GCS scores, TBIs were severe in 42.2%, moderate in 28.5%, and mild in 11.1%. In another study (J. Leitgeb, 2013) [23], the following pattern was observed: 57% had GCS scores of 13–15, 19% had scores of 10–12, 9% had scores of 7–9, and 15% had scores of 3–6 upon admission. The author stated that a low GCS score is more likely to produce unfavorable outcomes.

Our finding that patients with intraventricular hemorrhages have worse prognosis and more of them were placed in rehabilitation centers is going in line with the fact that traumatic intraventricular hemorrhage is associated with poor outcome; however, the difference in our study is that intraventricular hemorrhages were more in our study population [24].

In addition to that, acute subdural hematomas remain as a strong challenge for neurosurgeons, despite all advances in medical and surgical treatment, where less favorable outcome is still seen; even after decreasing mortality, subdural hematomas patients are prone to lower score on Glasgow outcome score, and they represent major portion of patients who need rehabilitation services when acute treatment is over [25].

The fact that almost 12.5% of our patients are undergoing craniectomy surgery reflects that our institution is believing in decompressive craniectomy and this may explain the reasonable low mortality; however [26] increase in number of patients who are going to rehabilitation hospitals or long-term care facilities is 34%. These findings reflect the need of rehabilitation center in almost every province in the Kingdom in the presence of MVA as a major national problem.

Since 2010, strict speed rules and regulations were implemented and cameras are now installed within cities and on highways, however, it took few years to cover the whole country. In 2018, new rules of forbidden text and drive and issuing tickets for such attitude are implemented and are expected to lower the occurrences of devastating car accidents. The impact of such regulations is worth reviewing in the next few years and compare to the current numbers.

Saudi Arabia is extremely concerned with the safety features in its imported vehicles, from all over the world, including airbags and ABS brakes systems. For the last 30 years, all cars have to go for the Periodic Inspection of Vehicle, which is electronically connected to car licensing authorities in Ministry of Interior (http://www.mvpi.com.sa). Saudi authorities have stopped importing any car older than 5 years since year 2010.

## 6. Limitations of the Study

The retrospective nature of the study and the lack of long-term follow-up of the patients and looking for the lifelong consequences like seizure disorders and psychiatric consequences are considered as one of the strongest limitations. The fact of missing some of the data is also considered as another limitation; however our study was the first in Aseer region that shed light on head injury burden, looked to the short-term outcomes, and addressed the fact that, despite the new traffic regulations, Aseer region still needs more attention to decrease the numbers of such devastating problems.

## 7. Conclusion

In this study, we observed that MVA is the leading cause of brain/head injuries in the KSA, despite the implementations of new speed rules. However, with new regulations of forbidding cell phone use while driving and forcing the seat belt regulations, a major impact on these numbers is expected in the future. Thus, a future study is recommended to assess these expectations.

## Figures and Tables

**Table 1:** Demographic variables (n=353).

Age (mean ± SD)	27.01 ± 13.9			
Gender	Male	Female		
	87.30%	12.70%		
Nationality	Saudi	Non-Saudi		
	94%	6%		
Residency	High	Low	Others	
	53.30%	38.30%	8.40%	
Job nature	Employed	Unemployed	Student	Worker
	42.50%	15.90%	34.6%	4%

**Table 2:** Categories of Glasgow coma score.

Head injury type based on GCS	Frequency	Percent
SEVERE (8 or less)	165	46.7

MODERATE (9-12)	149	42.2

MILD (13-15)	39	11.1

Total	353	100

GCS: Glasgow coma scale.

**Table 3:** Overall Glasgow outcome score in head injury patients.

Glasgow outcome scale:		
Scales (1-5)	Frequency	Percent
1	9	2.5

2	26	7.4

3	20	5.7

4	71	20.1

5	227	64.3

*Total*	*353*	*100.0*

Glasgow Outcome Scale GOS:

1- Dead.

2- Persistent vegetative/minimal responsiveness.

3- Severe disability/conscious but disabled; dependent on others for daily support.

4- Moderate disability/disabled but independent; can work in sheltered setting.

5- Good recovery/resumption of normal life despite minor deficits.

**Table 4:** Glasgow outcome based on type of head injury type.

Head injury type	Glasgow outcome score based					Total
	1	2	3	4	5	
EDH	4	4	2	19	52	81

SDH	0	6	8	10	40	64

SAH	2	6	5	12	47	72

Contusion	2	1	1	12	52	68

IVH	1	6	1	6	28	42

Fractures	0	2	2	6	16	26

Total	9	25	19	65	235	353

P = 0.136.

*EDH: epidural hematoma.*

*SDH: subdural hematoma.*

*SAH: subarachnoid hemorrhage.*

*IVH: intraventricular hemorrhage.*

**Table 5:** Comparison of type of head injuries with GCS scores.

GCS Score					
Head Injury		Severe	Moderate	Mild	Total
	EDH	24	27	33	81
	SDH	36	22	6	64
	SAH	15	57	0	72
	Contusion	43	20	5	68
	IVH	26	13	3	42
	Fractures	12	10	4	22

Total		165	149	39	353

Chi square = 7.815, d f = 2, and P <.05.

*EDH: epidural hematoma.*

*SDH: subdural hematoma.*

*SAH: subarachnoid hemorrhage.*

*IVH: intraventricular hemorrhage.*

**Table 6:** Comparison of Type of head injuries with outcome status.

Type of Head Injuries	Outcomes			Total
	Discharge	Refer to another center	Rehabilitation	
contusion	47	3	18	68

EDH	64	2	15	81

fractures	19	1	6	26

IVH	9	0	33	42

SAH	54	1	17	72

SDH	30	3	31	64

Total	223	10	120	353

Chi square = 9.5, d f = 2, and P <.05.

*EDH: epidural hematoma.*

*SDH: subdural hematoma.*

*SAH: subarachnoid hemorrhage.*

*IVH: intraventricular hemorrhage.*

**Table 7:** Comparison of type of head injuries with surgery types.

Head Injury Types	Head surgery type			Total
	Craniectomy	Craniotomy	No	
Contusion	2	0	66	68

EDH	3	32	46	81

fractures	1	3	22	26

IVH	1	0	41	42

SAH	21	0	51	72

SDH	16	0	48	64

Total	44	35	274	353

Chi square = 11.4, d f = 2, and P <.05.

*EDH: epidural hematoma.*

*SDH: subdural hematoma.*

*SAH: subarachnoid hemorrhage.*

*IVH: intraventricular hemorrhage.*

## Data Availability

The data used to support the findings of this study are available from the corresponding author upon request.
